# A New ABCB1 Inhibitor Enhances the Anticancer Effect of Doxorubicin in Both In Vitro and In Vivo Models of NSCLC

**DOI:** 10.3390/ijms24020989

**Published:** 2023-01-04

**Authors:** Maria Pia Adorni, Maricla Galetti, Silvia La Monica, Matteo Incerti, Alessandro Ruffoni, Lisa Elviri, Ilaria Zanotti, Bianca Papotti, Delia Cavallo, Roberta Alfieri, Pier Giorgio Petronini, Franco Bernini

**Affiliations:** 1 Department of Medicine and Surgery, University of Parma, 43125 Parma, Italy; 2 Department of Occupational and Environmental Medicine, Epidemiology and Hygiene, INAIL—Italian Workers’ Compensation Authority, Monte Porzio Catone, 00078 Rome, Italy; 3 Department of Food and Drug, University of Parma, 43124 Parma, Italy; 4 Institute of Organic Chemistry, RWTH Aachen University, Landoltweg 1, 52056 Aachen, Germany

**Keywords:** multidrug resistance (MDR), ATP-binding cassette B1 (ABCB1), lung cancer, NSCLC, doxorubicin

## Abstract

In tumors, the multi drug resistance phenomenon may occur through the efflux of chemotherapeutic drugs out of cancer cells, impeding their accumulation, and eventually reducing their toxicity. This process is mediated by transporters overexpressed in the plasma membranes of tumor cells, among which is the P-glycoprotein/multidrug resistance 1/ATP-binding cassette B1 (P-gp/MDR1/ABCB1). The aim of this study was to explore the effect of a new molecule, called AIF-1, on ABCB1 activity. In a cellular model of non-small cell lung cancer (NSCLC), AIF-1 significantly inhibited ABCB1 activity, which was evaluated by the fluorimetric measurement of the intracellular accumulation of calcein. AIF-1 also significantly increased the intracellular content of doxorubicin, which was evaluated by confocal microscopy and LC-MS/MS analysis. This effect translated to higher cytotoxicity of doxorubicin and reduced cellular proliferation. Finally, in a murine xenograft model, the tumor volume increased by 267% and 148% on average in mice treated with vehicle and doxorubicin alone, respectively. After the co-administration of doxorubicin with AIF-1, tumor volume increased by only 13.4%. In conclusion, these results suggest enhancement of the efficacy of the chemotherapeutic drug doxorubicin by AIF-1, laying the basis for the future development of new ABCB1 inhibitors for tumor treatment.

## 1. Introduction

Multidrug resistance (MDR) consists in a biological process that limits the efficacy of several pharmacological treatments, including chemotherapy. Some tumors may be intrinsically resistant to pharmacological therapy, even without exposure to cytotoxic drugs, whereas others, initially sensitive to chemotherapeutic agents, become resistant after long-term treatment [1]. Drug resistance can be mediated by a reduced level of the drug in tumor cells as a result of a poor uptake, increased metabolism, or enhanced efflux. The latter is mediated by transporters expressed in the plasma membrane which promote the drug’s extrusion from tumor cells, counteracting the intracellular accumulation of anticancer drugs, limiting their toxic effects, and eventually, their therapeutic efficacy [2]. P-glycoprotein/multidrug resistance 1/adenosine triphosphate-binding cassette (ABC) B1 (P-gp/MDR1/ABCB1), breast cancer resistant protein (BCRP, also known as ABCG2), and MDR-associated protein MRP1 (ABCC1), belonging to the ABC superfamily, are the most important efflux pumps involved in the MDR phenomenon [3]. Among these, ABCB1 is a consistently over-expressed transporter in drug-resistant tumors [4]. It is a 170 KDa phosphorylated glycopeptide encoded by *MDR1* gene and expressed in normal tissues, but it is highly overexpressed in several tumor cells [5], where it actively extrudes chemically, pharmacologically, and structurally unrelated anticancer drugs such as paclitaxel, doxorubicin, and vincristine [6,7]. Interestingly, a number of pieces of evidence support a correlation between the expression of this pump and tumor malignancy [2]. Therefore, inhibition of ABCB1 expression or activity could increase the accumulation of anticancer drugs within the cancer cells, enhancing their cytotoxicity and restoring their efficacy. Over the past three decades, four generations of ABCB1 inhibitors have been developed. Of these, a number of ABCB1 inhibitors entered clinical trials but had limited therapeutic success due to different issues, among which were side-effects [8]. Given the evidence of ABCB1-mediated limiting of drug accumulation and efficacy, the associations between the ABCB1 transporter and poor clinical outcomes, and the high levels of ABCB1 in patients whose tumors display drug resistance [9], the search for new, potent, efficacious, selective, and minimally toxic ABCB1 inhibitors should be re-evaluated.

In our previous study, we showed that probucol, a lipid-lowering phenolic drug [10], specifically inhibits the activity of ABCA1 [11], an ABC membrane transporter involved in cholesterol efflux from cells. Based on this observation, a specific set of probucol derivatives and analogues (Schemes S1–S5 of Supplementary Materials), either commercially available or obtained by chemical synthesis, were examined for their ability to inhibit the activity of ABCB1 in cancer cells. This led to the discovery of AIF-1 as a potent selective inhibitor of ABCB1. Thus, the aim of this work was to perform a further extensive characterization of the ABCB1 inhibitory action of this new probucol diester derivative, AIF-1, and its potential to enhance the antitumor effect of doxorubicin using in vitro and in vivo models of non-small cell lung cancer (NSCLC).

## 2. Results

### 2.1. Effect of AIF-1 on Different ABC Transporters’ Activity

**Figure 1:**
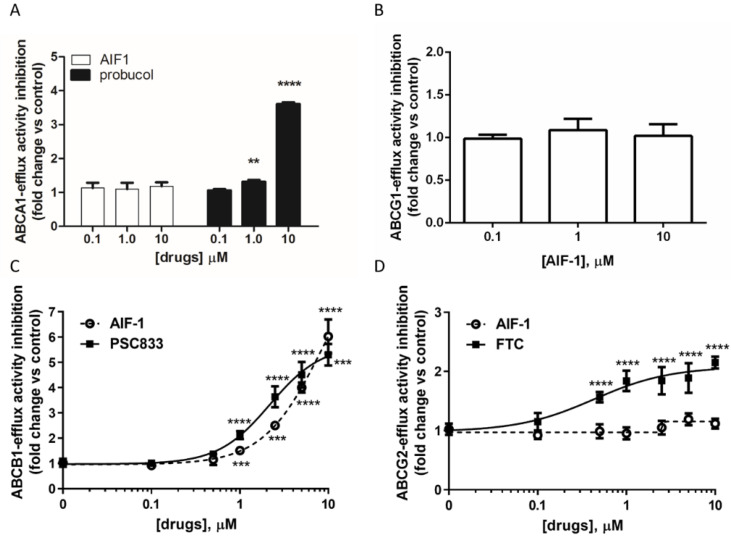
Characterization of AIF-1’s effects on different ATP-binding cassette transporters. (**A**) Effect on ABCA1 activity. Cholesterol efflux was evaluated in murine peritoneal macrophages (MPM), as described in Section 4. Relative ABCA1 activity was defined as the ratio of the percent of cholesterol efflux to apoA-I in untreated cells, and the percent obtained in cells treated with AIF-1 or probucol and is expressed as fold inhibition of activity compared to control. (**B**) Effect on ABCG1 activity. Cholesterol efflux was evaluated in Chinese hamster ovary cells, as described in Section 4. Relative ABCG1 activity was defined the ratio of percent of cholesterol efflux to HDL in untreated cells and the percent obtained in cells treated with AIF-1 and is expressed as fold inhibition of activity compared to control. (**C**) Effect on ABCB1 activity. Calcein AM fluorescence was determined in A549 cells as described in Section 4. Relative ABCB1 activity was defined as the ratio of calcein accumulation per microgram of protein between AIF-1 or PSC833-treated cells and untreated cells and is expressed as fold inhibition of activity compared to control. (**D**) Effect on ABCG2 activity. Hoechst 33342 fluorescence was determined in H460 cells by luminometer as described in Section 4. Relative ABCG2 activity was defined as the ratio of Hoechst 33342 accumulation per microgram of protein between AIF-1 or fumetrimorgin C-treated cells and untreated cells and is expressed as fold inhibition of activity compared to control. Statistical significance was calculated by ANOVA with Bonferroni’s post hoc analysis. Data are representative of at least three independent experiments with triplicate wells (*n* = 3). Values are expressed as mean ± SD. (** *p* < 0.01; *** *p* < 0.001; **** *p* < 0.0001 vs. control).

We first tested the ability of AIF-1 (compound **2** in Schemes S1–S5, Supplementary Materials) to modulate the activity of membrane transporters belonging to the ATP-binding cassette (ABC) family, which involved in the process of cholesterol efflux, namely, ABCA1 and ABCG1. As expected, probucol, the lead compound, significantly inhibited in a dose-dependent manner the ABCA1 activity, which was evaluated by cholesterol efflux to the cholesterol acceptor apolipoprotein A-I (apoA-I) from macrophages (Figure 1A). This effect was absent when the same cells were treated with the probucol derivative AIF-1 (Figure 1A). Moreover, in a specific cellular model overexpressing ABCG1 [12], cholesterol efflux to HDL, the parameter representing the activity of ABCG1, did not change after treatment with AIF-1 at all concentrations tested as compared to untreated cells, indicating the absence of any effect by our compound on this transporter (Figure 1B).

We then evaluated the effect of AIF-1 on the ABC transporters involved in drug extrusion, namely, ABCB1 and ABCG2. In the NSCLC A549 cell line expressing a high level of ABCB1 (Figure S1, Supplementary Materials), we could confirm that AIF-1 significantly inhibits ABCB1 activity, which was evaluated through intracellular accumulation of calcein (Figure 1C). In addition, this effect occurred in a dose-dependent manner. In this experimental condition, cells treated with 10 µM AIF-1 showed up to 6-fold-increased intracellular calcein-derived fluorescence with respect to control cells (IC_50_ = 8.6 μM). This result was in line with the observed increased intracellular calcein-derived fluorescence in A549 cells treated with the known ABCB1-specific inhibitor PSC833 (up to 5-fold with respect to control cells) (Figure 1C).

Finally, we investigated whether AIF-1 affects the activity of the ABCG2 transporter. Incubation with increasing concentrations of AIF-1 did not change intracellular Hoechst 33342-derived fluorescence in the NSCLC H460 cell line expressing high levels of this transporter [13], indicating a lack of a modulatory effect on the ABCG2 protein by our compound (Figure 1C). Conversely, the reference compound fumetrimorgin C, as expected, significantly inhibited the amount of Hoechst 33342-caused intracellular fluorescence (Figure 1D).

### 2.2. Comparison of AIF-1’s Effect on ABCB1 Activity in Cancer and Normal Cells

**Figure 2:**
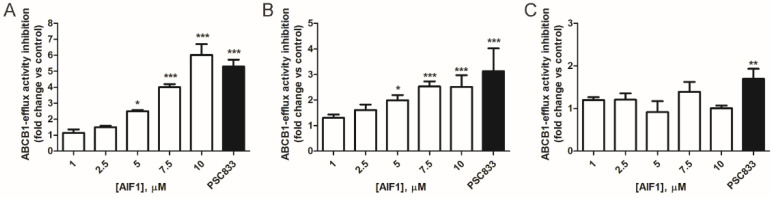
Effect of AIF-1 on ABCB1 activity in cancer and normal cells. (**A**) A549, (**B**) SKMES-1, and (**C**) BEAS 2B. Calcein AM fluorescence was determined by luminometer as described in Section 4. Relative ABCB1 activity was defined as the ratio of calcein accumulation per microgram of protein for AIF-1 or PSC833-treated cells to that of untreated cells and is expressed as fold inhibition of the activity compared to control. Results are representative of at least three independent experiments. Statistical significance was calculated by one-way ANOVA with Bonferroni’s post hoc analysis. Values are expressed as mean ± SD. (* *p* < 0.05, ** *p* < 0.01, *** *p* < 0.001 vs. ctr).

To further evaluate the ability of AIF-1 to inhibit ABCB1 activity and increase intracellular calcein content in tumor cells, we tested AIF-1 at concentrations between 1 and 10 µM in A549 and in a NSCLC cell line (SKMES-1) derived from squamous cell lung carcinoma (SQCLC), which expresses a high level of ABCB1 (Figure S1). After 4 h of incubation with calcein AM in the presence of AIF-1, A549 cells showed a dose-dependent accumulation of intracellular fluorescence calcein (Figure 2A). This effect was also confirmed in the squamous cell line SKMES-1, and it occurred in a dose-dependent manner (Figure 2B).

Interestingly, when we evaluated intracellular calcein content in a normal human immortalized lung epithelial cell line (BEAS 2B), AIF-1 did not affect intracellular calcein accumulation (Figure 2C).

To further investigate the underlying mechanism of AIF-1 activity on ABCB1, we studied in SKMES-1 cells the accumulation of the ABCB1 substrate calcein at different concentrations (0.5, 1, 2.5, 5, 10, and 20 µM) in the presence of AIF-1 or the reference compound PSC833. The resulting retention rates of substrate are displayed in the Lineweaver–Burk double-reciprocal plots (Figure S2, Supplementary Materials). As previously reported [14,15], PSC833 inhibited ABCB1-mediated calcein transport in a non-competitive manner. Similarly, the obtained regression curves suggested a non-competitive ABCB1 inhibition mechanism mediated by AIF-1.

### 2.3. In Vitro Evaluation of AIF-1 Cytotoxicity

**Figure 3:**
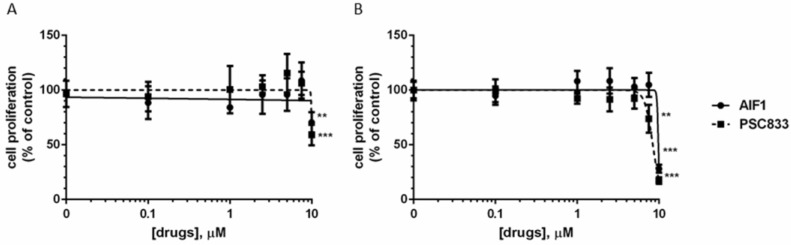
Effect of AIF-1 on cell proliferation. BEAS 2B (**A**) and SKMES-1 (**B**) cells were treated with AIF-1 or PSC833. After 72 h, a crystal violet assay was performed, and absorbance was measured at 570 nm. Data are expressed as percentages of cell growth versus control cells. Results are representative of at least three independent experiments. Statistical significance was calculated by one-way ANOVA with Bonferroni’s post hoc analysis. Values are expressed as mean ± SD. (** *p* < 0.01, *** *p* < 0.001 vs. ctr).

We next evaluated the potential cytotoxicity of AIF-1 in both normal and cancer cells. To this end, we exposed BEAS 2B (Figure 3A) and SKMES-1 cells (Figure 3B) to increasing concentrations of AIF-1 and PSC833 (separately), evaluating their effects on cell proliferation by crystal violet assay. After 72 h, at concentrations of AIF-1 and PSC833 of up to 7.5 µM, the cell growth rate never dropped below 85 ± 2% (vs. control) in both the cell lines. At the highest concentration (10 µM), we observed a reduced cell proliferation rate, although it was more pronounced in the presence of the reference compound, PSC8333. In this experiment, the half-maximal growth inhibition concentrations (GI50) of AIF-1 in BEAS 2B and SKMES-1 were >10 and 9.8 μM, respectively.

### 2.4. Effect of AIF-1 on Doxorubicin’s Intracellular Accumulation and Cytotoxicity

**Figure 4:**
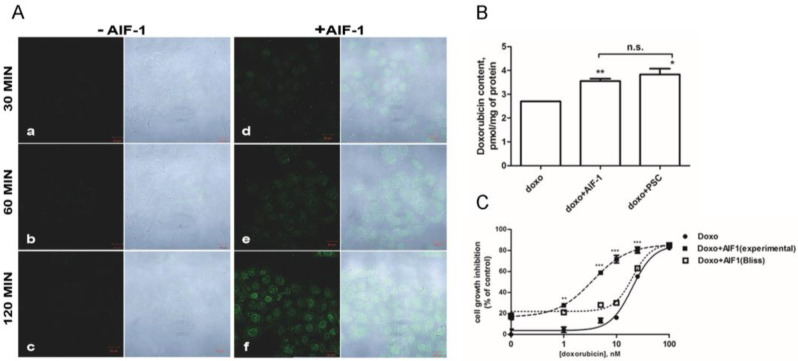
Effect of AIF-1 on intracellular doxorubicin content and doxorubicin cytotoxicity. (**A**) SKMES-1 cells were pre-treated with or without 10 µM AIF-1 for 30 min and subsequently incubated with 5 µM doxorubicin. After 30 (**a**,**d**), 60 (**b**,**e**), and 120 min (**c**,**f**), the doxorubicin-derived fluorescent signal (green fluorescence) was acquired with a confocal microscope; scale bar 20 µm. The same field is shown by light transmission images in all the panels (white panels). Images are from a representative experiment; each experiment, repeated three times, yielded similar results. (**B**) SKMES-1 cells were exposed to 50 nM doxorubicin for 24 h (with or without 7.5 µM AIF-1 or PSC833), and the amount of doxorubicin was quantified by LC-MS/MS analysis. Doxorubicin levels are expressed as pmol/mg of protein, and values given are the means (±SD) of three independent determinations. Statistical significance was calculated by Student’s *t*-test (* *p* < 0.05; ** *p* < 0.01; n.s.: not significant). (**C**) Curves of growth-inhibitory effects of various concentrations of doxorubicin and the combined treatment with 5 µM AIF-1 (experimental) versus the theoretical Bliss additivity curve (Bliss). Cells were counted after 72 h, and data are expressed as percent inhibition of cell growth versus control cells. Statistical significance was calculated by Student’s *t*-test. The experiments, repeated three times, yielded similar results. (** *p* < 0.01; *** *p* < 0.001).

We investigated whether AIF-1 treatment affects the intracellular content of doxorubicin, a well-known ABCB1 substrate [16]. We thus evaluated doxorubicin-derived intracellular fluorescence after treatment with AIF-1 by using confocal microscopy. SKMES-1 cells were pre-treated or not (control cells) with AIF-1 for 30 min and subsequently incubated with doxorubicin. Doxorubicin-derived fluorescence was acquired at different time points (30, 60, and 120 min). Pre-treatment with AIF-1 promoted a greater increase in the doxorubicin-derived fluorescent signal in a time-dependent manner (Figure 4A(d–f)), especially in the perinuclear and nuclear regions of cells. In contrast, no fluorescence was observed in control cells at any time (Figure 4A(a–c)).

We then used liquid chromatography–tandem mass spectrometry analysis for a direct quantitative determination of the doxorubicin amount within cancer cells after treatment with AIF-1. Pre-treatment with AIF-1 was actually able to increase the intracellular content of doxorubicin in SKMES-1 cancer cells (from 2.70 ± 0.01 to 3.55 ± 0.10 pmol/mg protein, *p* = 0.0069) to a similar extent as the reference compound, PSC833 (from 2.70 ± 0.01 to 3.83 ± 0.24 pmol/mg protein, *p* = 0.0219) (Figure 4B).

Considering that simultaneous treatment with doxorubicin and AIF-1 increased the intracellular doxorubicin content, we evaluated the effect of the drug combination on cell proliferation. SKMES-1 cells were treated for 72 h with various concentrations of doxorubicin in the presence or absence of AIF-1. As shown in Figure 4C, in the presence of AIF-1, the GI_50_ of doxorubicin was reduced by about eight times. In addition, when comparing the experimental combination points with that expected by the Bliss criterion, a synergistic effect of the two drugs on growth inhibition can be observed for SKMES-1 at all the tested doxorubicin concentrations (Figure 4C). Similar results were obtained when we tested the ability of AIF-1 to reverse ABCB1-mediated MDR, using various concentrations of AIF-1 (5 and 7.5 μM) (Figure S3A, Supplementary Materials). The results confirmed that the EC_50_ of doxorubicin was reduced by approximately 6-fold in the presence of AIF-1, as shown by the shift to the left of the concentration-effect curve, and thus, less doxorubicin is required for half-maximal growth inhibition in the presence of compound AIF-1. Finally, we assessed the time-dependence of the efficacy of AIF-1 combined with 10 nM doxorubicin. The maximum effect induced by the combined treatment was after 3 days of treatment, and no further effects were observed on subsequent days (Figure S3, panel B, Supplementary Materials). 

### 2.5. Effect of AIF-1 on the Antitumor Efficacy of Doxorubicin In Vivo

**Figure 5:**
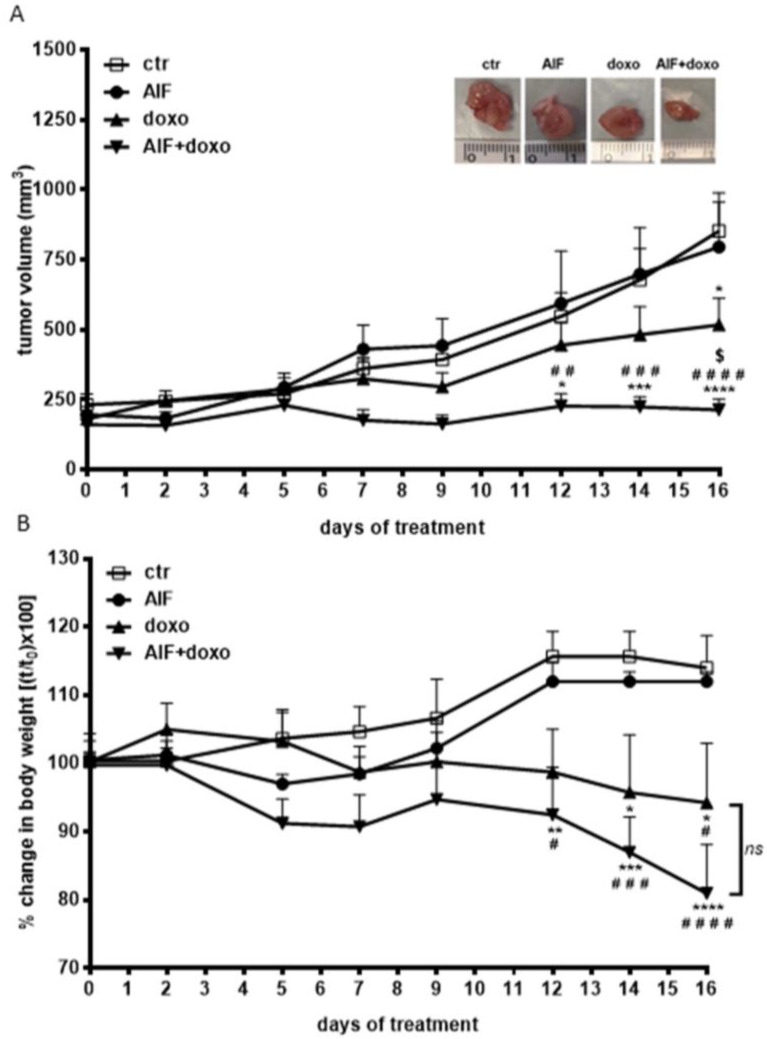
Effect of AIF-1 and doxorubicin treatment on a SKMES-1 tumor xenograft. (**A**) SKMES-1 cells were implanted s.c. on BALB/c-nude mice, and after tumors had reached an average size of approximately 200 mm^3^, the animals were randomized into four different groups. AIF-1 (25 mg/kg) was administered s.c. every day, and doxorubicin (4 mg/kg) was administered i.p. once a day, three times per week. Tumor sizes were measured two times per week, and data are expressed as mean tumor volume ± SEM of 8 tumors (* *p*< 0.05, *** *p* < 0.001, **** *p* < 0.0001 vs. ctr; *## p <* 0.01; ### *p* < 0.001, #### *p* < 0.0001 vs. AIF; $ *p* < 0.05 vs. doxorubicin; two-way repeated measures analysis of variance followed by Bonferroni’s post-test). (Inset) Representative images of dissected xenograft tumors. (**B**) At the indicated time points, percentage change in body weight was calculated in all mice as (weight on day x/weight on day zero) × 100, and data are expressed as mean ± SEM (*n* = 8). * *p* < 0.05, ** *p* < 0.01, *** *p* < 0.001, **** *p* < 0.0001 vs. ctr; # *p* < 0.05, ### *p* < 0.001, #### *p* < 0.0001 vs. AIF; ns: not significant; two-way repeated-measures analysis of variance followed by Bonferroni’s post-test.

Based on the in vitro findings, we investigated whether AIF-1 could enhance the antitumor effect of doxorubicin in an in vivo model of NSCLC. To this end, AIF-1 preparation at the gram scale and optimization of the reaction yield were performed (see Supplementary Materials). SKMES-1 cells were subcutaneously injected into BALB/c nude female mice, and when tumors reached an average volume of about 200 mm^3^, animals were randomly assigned into four groups: vehicle (ctr), AIF-1 (AIF-1, 25 mg/kg subcutaneously), doxorubicin (doxo, 2 mg/kg, intraperitoneally), and the combination of AIF-1 and doxorubicin (AIF-1+doxo). After 16 days of treatment, the means of tumor volume were 854.48 ± 135.84, 796.08 ± 160.52, 517.16 ± 95.46, and 214.05 ± 37.61 mm^3^ in ctr, AIF-1, doxo, and AIF-1+doxo groups, respectively (Figure 5A).

The doxorubicin-alone group showed a statistically significant decrease in tumor volume compared to ctr only at day 16 (−39.5%, *p* < 0.05) (Figure 5A). When doxorubicin was administered in association with AIF-1, its antitumor effect was significantly potentiated, as the drug combination completely blocked the growth of the tumor, keeping the volume very close to the original size at all-time points. The difference in tumor volume compared to the control group was statistically significant starting from the 12th day of treatment. At the end of the study (day 16), the tumor volume was 74% lower after treatment with the combination of the two compounds (*p* < 0.0001) compared to ctr and 58.6% lower (*p* < 0.05) compared to the doxorubicin-alone group. AIF-1 alone did not affect tumor volume. 

To evaluate whether this potentiating effect of AIF-1 could be related to interference with doxorobucin’s pharmacokinetics, the plasma concentration of doxorubicin was determined at the end of the treatments, showing no significant differences between doxo and AIF-1+doxo groups (doxo, 3.09 ± 1.24 µg/mL; AIF-1+doxo, 3.99 ± 0.79 µg/mL, *p* = 0.345, ns).

As expected, doxorubicin-treated xenografts exhibited signs of distress and rapid weight loss [17]. On the contrary, AIF-1 did not induce any toxicity alone or in combination with doxorubicin, as evidenced by the animal weights, which did not differ significantly between control and AIF-1, or between doxorubicin and doxorubicin in combination with AIF-1 (Figure 5B). 

## 3. Discussion

In this study, we identified a synthetic compound called AIF-1 as a novel modulator of the ATP-binding cassette B1 (ABCB1) protein, the most studied among the membrane pumps involved in the resistance to chemotherapeutic drugs. It limits their clinical efficacy [18]. We demonstrated that AIF-1 enhanced the sensitivity of NSCLC cells to the toxic effect of doxorubicin as a consequence of the inhibition of the efflux function of ABCB1, and consistently, we showed that the co-administration of AIF-1 with doxorubicin in a model of xenograft inhibited tumor growth without further toxicity compared to doxorubicin alone.

The new compound, AIF-1, is a disuccinic ester derived from probucol (see Schemes S1–S5, Supplementary Materials), a diphenolic compound with lipid-lowering activity that was demonstrated to be a specific inhibitor of ABCA1, a membrane transporter involved in cholesterol efflux from cells [11,19].

We demonstrated that by modifying probucol’s chemical structure by esterification of the two phenolic functional groups with succinic-free carboxylic acid, an ABCA1 inhibitor can be converted into an ABCB1 inhibitor. In this regard, calcein AM is a well-known substrate of ABCB1 [20,21,22], and using a functional test based on measurement of its intracellular accumulation [23], we could demonstrate that AIF-1 significantly induced calcein accumulation in NSCLC cellular models, an effect likely related to the inhibition of the calcein efflux process mediated by the membrane pump. 

With respect to the mechanism of action, the kinetic studies of the binding mode of AIF-1 showed that this compound possibly established non-competitive inhibition of the substrate calcein in the binding site of ABCB1, similarly to PSC833 [14,15].

Importantly, among the tested transporters, AIF-1 was more selective for ABCB1, as it did not show any effects on the activity of ABC transporters involved in cell cholesterol efflux, i.e., ABCA1 and ABCG1 [24], nor on ABCG2, which is involved in MDR. 

Moreover, AIF-1 was inactive in modulating intracellular calcein content in normal human immortalized lung epithelial cell line (BEAS 2B), a widespread standardized model for pulmonary epithelial function expressing functional ABCB1 [25], suggesting a greater sensitivity of cancer cells to the action of AIF-1 as compared to normal cells. 

Doxorubicin belongs to the anthracycline drug family and is included in the current adjuvant chemotherapeutic regimens in the treatment of lymphomas, sarcomas, and a broad spectrum of solid tumors, including breast, lung, bladder, bone, and cervical ones [26,27]. Although considered as a first-line drug for various types of cancers, one of the main obstacles to doxorubicin therapy is drug resistance [28], which is mainly mediated by ABCB1, for which doxorubicin represents a good substrate [29,30]. Interestingly, it was reported that treatment with doxorubicin induced a large increase in ABCB1 expression in lung cancer cells, whereas no significant change in expression was observed in normal lung cells [31]. It is well known that the increased cellular expression of this transporter protects tumor cells from the effects of toxins, including chemotherapeutic drugs. Therefore, the use of ABCB1 inhibitors might represent a good strategy to overcome the cell efflux of chemotherapeutic drugs improving the clinical outcomes [2,32]. 

In this study, we provided evidence that AIF-1 increases the accumulation of doxorubicin within lung cancer cells, leading to enhanced cytotoxicity of doxorubicin. Using confocal laser scanning fluorescent microscopy, we demonstrated that pre-treatment with AIF-1 increased doxorubicin’s fluorescence in the perinuclear and nuclear regions of cells, an effect likely due to the impact of the compound on ABCB1. Consistently, similar results were obtained when cells were pre-treated with PSC833, a well-known ABCB1 inhibitor [15]. These observations are consistent with those previously reported by Heibein and colleagues, although in a different cellular model, suggesting that doxorubicin can better reach its target, the nuclear DNA, only when cells are co-treated with an ABCB1 inhibitor [33]. Moreover, the results obtained by confocal microscopy were completely consistent with our finding that AIF-1 increased the amount of anthracycline in tumor cells, which was measured directly by high-performance liquid chromatography. Together, these observations suggest that the differential fluorescence localization in the absence or presence of AIF-1 (diffuse vs. concentrated in the nuclear region) may be directly related to the marked accumulation of doxorubicin when used with AIF-1, which in turn might lead to an increase in doxorubicin’s anti-cancer effect. This hypothesis was confirmed by the finding that the combined treatment significantly increased the sensitivity of tumor cells to the toxic effect of anthracycline.

The combined effects of drugs are deeply studied through the Bliss independence criterion for in vitro co-exposure [34]. We showed synergistic growth inhibition associated with a significantly marked reduction in the GI_50_ of doxorubicin when used in combination with AIF-1. Since it was previously reported that concentration- and time-dependent assays could implement MDR reversal analysis, providing new measures for a more detailed analysis of the efficacy of a compound [35], we also studied these parameters. The analysis showed that this compound enhanced the cytotoxicity of doxorubicin in a dose-dependent manner, even if the time-dependently increased was observed only after 3 days of treatment, and no further effects were observed on subsequent days. 

Further, it appears that the co-incubation of doxorubicin with AIF-1 not only improves the efficacy of the antineoplastic agent by favoring its intracellular accumulation, but also potentiates its effect. Indeed, intracellular doxorubicin content increases not only after PSC833 treatment, but also after AIF-1 treatment, making cells more sensitive to drug treatment.

Finally, our results indicate that combined treatment with doxorubicin and AIF-1 exerted a cytostatic and not a cytotoxic effect, involving suppression of proliferation rather than enhancement of cell death. Indeed, we did not find a significant increase in the rate of cells death at all the tested conditions.

Our in vitro results were confirmed by in vivo experiments using a murine model with a xenograft. This approach allowed us to prove that AIF-1 significantly increased the antitumor activity of doxorubicin. Indeed, its combination with AIF-1 completely abolished the tumor growth, stabilizing the volume at values close to those of baseline. Doxorubicin alone did not prevent tumor growth, having only a partial inhibitory effect observed on the last day of treatment, indicating resistance to the toxic effect of the drug by cancer cells. These data confirm that AIF-1 is able to enhance sensitivity of lung cancer cells towards doxorubicin in an in vivo model. Importantly, our in vivo results showed the absence of additional AIF-1-related toxicity measured as mouse body weight with respect to doxorubicin alone. Moreover, we showed that AIF-1 at the dose studied did not affect the plasma concentration of doxorubicin, ruling out reduced clearance of doxorubicin induced by the compound. This result indicates that the observed increased anti-tumoral effect of doxorubicin induced by AIF-1 is not attributable to the sustained presence of the anticancer drug in the circulation.

The presence of “endogenous or physiological” ABCB1 protein has become problematic to the chemical inhibition strategy. Indeed, ABCB1 is expressed in barrier tissues to sanctuary sites (e.g., the blood–brain barrier) and in secretory/absorptive tissues (e.g., renal tubules and the gastrointestinal tract) [36]. At these sites, the protein acts as a cellular defender, and it contributes to the overall pharmacokinetic profiles of numerous drugs, including chemotherapeutic drugs. Therefore, even with our new compound, the appearance of side-effects needs to be explored in an ad hoc study; the observed relative selectivity of the action of the new compound AIF-1 might be of potential clinical advantage. 

## 4. Materials and Methods

### 4.1. Cell Culture

The human non-small cell lung cancer (NSCLC) cell lines H460, SKMES-1, and A549; and the human bronchial epithelial cells BEAS 2B were cultured in RPMI-640 containing 2 mM glutamine, 100 U/mL penicillin/100 μg/mL streptomycin, and 10% fetal bovine serum (Life Technologies, Carlsbad, CA, USA). Mouse peritoneal macrophages, harvested from the peritoneum of C57 b/6 mice, as previously described [37], were used for the valuation of ABCA1 activity and were cultured in DMEM (Lonza, Verviers, Belgium) in the presence of antibiotics (penicillin–streptomycin, Thermo Fisher Scientific, Waltham, MA, USA) and supplemented with 10% fetal calf serum (FCS) (Sigma Aldrich, St. Louis, MO, USA). Chinese hamster ovary cells (CHO) transfected or not with the human abcg1 gene were used for the evaluation of ABCG1 activity and were cultured in Ham’s F-12 (Lonza Bioscience, Basel, Switzerland) in the presence of antibiotics (Zeocin and Geneticin, Thermo Fisher Scientific) and 10%FCS. Cell lines were from American Type Culture Collection (Manassas, VA, USA) and were maintained under standard cell culture conditions at 37 °C in a water-saturated atmosphere of 5% CO_2_ in air.

### 4.2. Compounds

In all assays, the tested compounds were dissolved in DMSO immediately before their addition to culture media. The final concentration of DMSO never exceeded 0.1% (*v*/*v*), and an equal amount of the solvent was added to control cells in basal conditions (no treatment). The ABCA1 and ABCG2 inhibitors, probucol and fumetrimorgin C, were purchased from Sigma Aldrich (St. Louis, MO, USA); the ABCB1 inhibitor PSC833 was kindly provided by Novartis. AIF-1 was synthetized from probucol, as described in the Supplementary Materials.

### 4.3. Flow Cytometry

One million cells were incubated with isotype-controlled monoclonal mouse IgG2/R-PE clone MPC-11 (Ancell IRP, Bayport, MN, USA) or PE mouse anti-human ABCB1 (BioLegend, London, UK) to determine ABCB1 protein membrane levels, as previously described [38]. After one hour of incubation, the analysis was performed using a Beckman-Coulter EPICS-XL flow cytometer. Mean fluorescence intensity (MFI) values were converted into units of equivalent fluorochrome (MEF) using the FluoroSpheres 6-Peak Kit (Dako, Carpinteria, CA, USA).

### 4.4. ABCs’ Function

To evaluate the functionality of the ABCG2 protein, we used the Hoechst 33342 dye accumulation assay, performed as previously described [13], whereas to assess the functionality of the ABCB1 protein, we used the calcein acetoxymethyl ester (calcein AM) assay. Calcein AM is a non-fluorescent, highly lipid-soluble dye that can rapidly penetrate the plasma membranes of normal cells. Once inside the cell, ester bonds are cleaved by endogenous esterases, transforming calcein AM into the hydrophilic and intensely fluorescent calcein. In normal cells, calcein is well retained in the cytosol, whereas in MDR cells expressing high levels of ABCB1, non-fluorescent calcein AM is rapidly extruded from the plasma membrane, reducing the accumulation of fluorescent calcein in the cytosol. The degree of inhibition of ABCB1 activity can be quantitated by measuring the increase in intracellular calcein fluorescence. Modulators of the transporter activity reduce the rate of calcein AM efflux, leading to increased intracellular calcein AM, which is then hydrolyzed by intracellular esterases, making it fluorescent [39]. Cells were plated at a density of 10,000 cells/well in 96-well plates (Perkin Elmer, Boston, MA, USA) and incubated for 48 h in complete growth medium at 37 °C. After removing the medium, cells were incubated in phenol red-free medium with 1µM calcein AM substrate for 4 h at 37 °C, in the presence or absence of the inhibitors PSC833 or AIF-1. Then, cells were washed twice with ice-cold PBS, and dye accumulation was measured in a fluorescence spectrophotometer (EnSpire Multimode Plate Readers, Perkin Elmer, Boston, MA, USA) at 485 nm (excitation)/535 nm (emission) for calcein. Trichloroacetic acid (TCA, 5%, *w*/*v*) was then added to precipitate cell proteins, which were further dissolved in 0.5 N NaOH, and their concentrations were determined by a dye-fixation method (Bio-Rad, Hercules, CA, USA) using bovine serum albumin as a standard [40]. The results were expressed as absorbance 485 nm/535 nm, per microgram of protein. Relative ABCB1 activity was defined as the ratio of calcein accumulation per microgram of protein between PSC833/AIF-1 treated cells and untreated cells.

To evaluate the functionality of ABCA1 transporter, we measured the cholesterol efflux process from murine macrophages by a radioisotopic technique, as previously described [41]. Briefly, murine peritoneal macrophages (MPM) were seeded in 24-well plates and then labeled with 2 µCi/mL [1,2-^3^H] cholesterol for 24 h. Cells were subsequently incubated overnight in medium containing 0.2% bovine serum albumin (BSA) (Sigma Aldrich, Milan, Italy) with or without 5 µg/mL 22-hydroxycholesterol (22-OH)/10 µmol/L 9-cis retinoic acid (9cRA) (Sigma Aldrich) to upregulate ABCA1. Efflux was measured by the apolipoprotein A-I (apoA-I), the specific cholesterol acceptor for the ABCA1 transporter, after 2 h incubation of cells with probucol, the specific ABCA1 inhibitor [11], or AIF-1, at the concentrations indicated. Relative ABCA1 activity was defined as the ratio of percentage efflux of cholesterol to apoA-I in untreated cells and that obtained in cells treated with AIF-1 or probucol.

To evaluate the functionality of ABCG1 transporters, we measured the cholesterol efflux process using a radioisotopic technique as previously described [42]. Briefly, Chinese hamster ovary cells (CHO) were seeded in 24-well plates for 24 h and labeled with 1 µCi/mL [1,2-^3^H] cholesterol for the following 24 h. Cells were subsequently incubated for 90 min in medium containing 0.2% (BSA) (Sigma Aldrich) in the presence of AIF-1 at the concentrations indicated. Efflux of high density lipoprotein (HDL) was then promoted, the specific cholesterol acceptor for the ABCG1 transporter. Cholesterol efflux was calculated as the percentage (%) of radiolabeled cholesterol released into the medium of the total radioactivity of the cells.

### 4.5. Determination of Cell Proliferation

Cell proliferation was evaluated by counting the cells in a Bürker hemocytometer with the trypan blue exclusion method and by crystal violet staining, as previously described [43]. The nature of the interaction between doxorubicin and AIF-1 was calculated using the Bliss interaction model [34,44]. A theoretical dose–response curve was calculated for combined inhibition using the equation Ebliss = EA + EB − EA × EB, where EA and EB are the percentages of inhibition versus control cells, obtained by drug A (doxorubicin) and B (AIF-1) alone; and Ebliss is the percentage of inhibition that would be expected if the combination was exactly additive. If the experimental percent of inhibition is >Ebliss, the combination is considered synergistic; if it is <Ebliss, the combination is antagonistic.

### 4.6. Immunofluorescence and Confocal Microscopy

SKMES-1 cells were seeded on coverslips in six-well plates, and they were allowed to grow overnight. On the following day, the coverslips were mounted in a microscope chamber, lodged inside a micro-incubator where culture conditions were kept constant (37 °C temperature, 5% CO_2_), in order to obtain images of living cells [45]. The chambers were perfused sequentially with PBS and with medium with 5 µM doxorubicin. Serial images at 30 min intervals were collected and analyzed. To study the effects of AIF-1 on ABCB1 activity and doxorubicin accumulation, SKMES-1 cells grown on coverslips were pre-exposed to 10 µM AIF-1 for 30 min before doxorubicin treatment. Fluorescence was acquired with a Zeiss LSM510 Meta confocal microscope (CLSM 510) (Carl Zeiss, Jena, Germany) with a 63× oil objective. This microscope was integrated with the confocal system, a LSM 510 Meta scan head. Doxorubicin fluorescence was excited with an argon laser at 488 nm, and the emission was collected through a 530 nm long-pass filter. Image acquisition was carried out in multitrack mode, namely, through consecutive and independent optical pathways.

### 4.7. LC-MS/MS Analysis

The extract samples in ethanol were filtered by cellulose filters (0.2 µm) and analyzed by liquid chromatography–tandem mass spectrometry (LC-MS/MS).

The analyses were carried out by using a liquid chromatograph HP 1200 (Agilent Technologies, Palo Alto, CA, USA) equipped with an autosampler coupled with an electrospray interface (ESI) and a triple quadruple mass spectrometer QTrap 4000 (ABSCIEX, Framingham, MA, USA). The LC separation was obtained by using a Sunshell column C18 (75 mm × 2.1 mm, 2.6 µm) (ChromaNik Technologies, Osaka, Japan), thermostated at the temperature of 25 °C. The mobile phase consisted of a mixture (A) water and (B) acetonitrile, and the separation was performed under gradient elution as follows: 0–2 min 5% (B), 2–5 min 50% (B), 5–6 min 80% (B), 6–8 min 80% (B), 8–9 min 5% (B), 9–14 min 5% (B). The mobile phase’s flow rate was 0.2 mL/min; injection volume was 20 μL. The system was controlled by the Analyst v1.6 software (ABSCIEX). Source parameters were: capillary voltage 4.5 kV, source temperature 350 °C, declustering potential 70 V, entrance potential 10 V. The full-scan mass spectra were recorded in the 250–900 *m*/*z* range, using a step size of 0.1 and a scan time of 1 s in the positive ion mode for doxorubicin and atenolol (ATN, used as internal standard) and in negative ion mode for AIF-1. The mass fragmentation spectra for doxorubicin and AIF-1 were acquired in product ion scan mode. We selected as the precursor ion the deprotonated and protonated molecular ions, respectively, and recorded the signal in the *m*/*z* 50–800 range, with collisional energy (CE) between 5 and 100 eV. The validation method and the quantitative analysis were carried out in the selected reaction monitoring mode (SRM): doxorubicin *m*/*z* 544.5/397.3 (EC 18 eV), 544.5/379 (EC 30 eV); AIF-1 *m*/*z* 715.7/515.5 (EC −25 eV); ATN (IS) *m*/*z* 267.3/145.3 (EC 33 eV). The calibration curve was constructed in the 2.5–100 ng/m: (doxorubicin) and 10–1000 ng/mL (AIF-1) ranges.

### 4.8. Tumor Xenografts

A total of 5 × 10^6^ SKMES-1 cells were suspended in 200 μL of Matrigel (BD Biosciences, Erembodegem, Belgium) and PBS (1:1) and were subcutaneously injected in the flanks of Balb/c-nude female mice (Charles River Laboratories, Calco, Italy) [46]. The animals were housed in a protected unit for immunodeficient animals with 12 h light-dark cycles and provided with sterilized food and water ad libitum. When tumor volume reached an average size of 200 mm^3^, the animals were randomized into four groups (*n* = 8): control, AIF-1, doxorubicin, and AIF-1 + doxorubicin. Every day, AIF-1 was subcutaneously (s.c) administered at a dosage of 25 mg/kg in DMSO, whereas doxorubicin (4 mg/kg in 0.9% NaCl injection) was given intraperitoneally (i.p.) once a day, three times per week. Control mice received vehicle according to the same schedules. Tumor xenografts were measured as previously described [47]. After 16 days of treatment, mice were euthanized by cervical dislocation, and tumors were collected for HPLC analysis. All experiments involving animals and their care were performed with the approval of the Local Ethical Committee of University of Parma (Organismo per la Protezione e il Benessere degli Animali) and by the Italian Ministry of Health, in accordance with the institutional guidelines that are in compliance with national (D.Lgs. 26/2014) and international (Directive 2010/63/EU) laws and policies.

### 4.9. Statistical Analysis

Statistical analyses were carried out using GraphPad Prism version 5.00 software (GraphPad Software Inc., San Diego, CA, USA). Results are expressed as mean values ± standard deviations (SD) for the indicated numbers of independent measurements. IC_50_ and GI_50_ values, expressed as means ± SD of three independent determinations, were calculated by fitting the experimental data with a hyperbolic function and constraining Ymax to 100 for GI**_50_** calculation. The significance of differences between the mean values recorded for different experimental conditions was calculated by the Student’s *t*-test, and multiple comparison among groups was performed using analysis of variance (one-way ANOVA) followed by Bonferroni’s post-test. For in vivo studies, comparison among groups was performed using two-way repeated-measures ANOVA followed by Bonferroni’s post-test (to adjust for multiple comparisons). *p* values are indicated where appropriate in the figures and in their legends. Adjusted *p* values of less than 0.05 were considered significant.

## 5. Conclusions

In conclusion, by using both an in vitro and an in vivo approach, we showed that the newly synthetized phenolic compound AIF-1 is a potent ABCB1 modulator, as it inhibits the ABCB1-mediated resistance in NSCLC cells by increasing intracellular doxorubicin concentration and cancer-cell sensitivity to its toxicity. Consistently, AIF-1 was able to stabilize tumor volume in a xenograft animal model when co-administered with doxorubicin. The present data lay the groundwork for future development of new classes of modulators of the ABCB1 efflux pump with potential clinical relevance, as they can improve the efficacy of chemotherapy in resistant cancers.

## 6. Patents

European patent application EP 3 193 858 B1 entitled “New MDR1 inhibitors for overcoming multidrug resistance”. Concession date 31 October 2018.

## Data Availability

The authors declare that the data generated and analyzed during this study are included in this published article and associated Supplementary Materials. In addition, datasets generated and/or analyzed during the current study are available from the corresponding author on reasonable request.

## Supplementary Materials

The following are available online at https://www.mdpi.com/article/10.3390/ijms24020989/s1. References [48,49,50,51,52,53] are cited in supplementary materials.
