# Supplementary material for: A New ABCB1 Inhibitor Enhances the Anticancer Effect of Doxorubicin in Both In Vitro and In Vivo Models of NSCLC
Source: Int J Mol Sci. 2023 Jan 4;24(2):989. doi: 10.3390/ijms24020989 (PMC9861803; doi:10.3390/ijms24020989)
Supplement: Supplementary file 1 [file ijms-24-00989-s001.zip › ijms-2134346-supplementary.pdf]

Supplementary materials

Chemistry

Unless otherwise noted, reagents were obtained from commercial suppliers and were used without purification. Anhydrous tetrahydrofuran (THF) was obtained by distillation over Na/Benzophenone. Anhydrous dichloromethane was obtained by distillation over calcium hydride.

All reactions were carried out using flame-dried glassware under atmosphere of nitrogen. The progress of the reactions was monitored by thin layer chromatography with F_{254} silica-gel precoated sheets (Merck, Darmstadt, Germany). UV light was used for detection. Flash chromatography was performed using Merck silica gel 60 (Si 60, 40-63 μm, 230-400 mesh ASTM).

Mass spectra were recorded on an Applied Biosystem, API 150 EX LC/MS system spectrometer.

^{1}H spectra were recorded on a Bruker Avance 400 spectrometer. Spectra were acquired from samples as CDCl3: (10 mg mL^{-1}). ^{1}H spectra (400.13 MHz) were measured at 25 °C in 5 mm o.d. tubes. Chemical shifts are reported as δ (ppm); coupling constants (*J*) are expressed in Hz.

Compounds **1–10** (Schemes S1–S5) were synthesized according to the procedures described below.

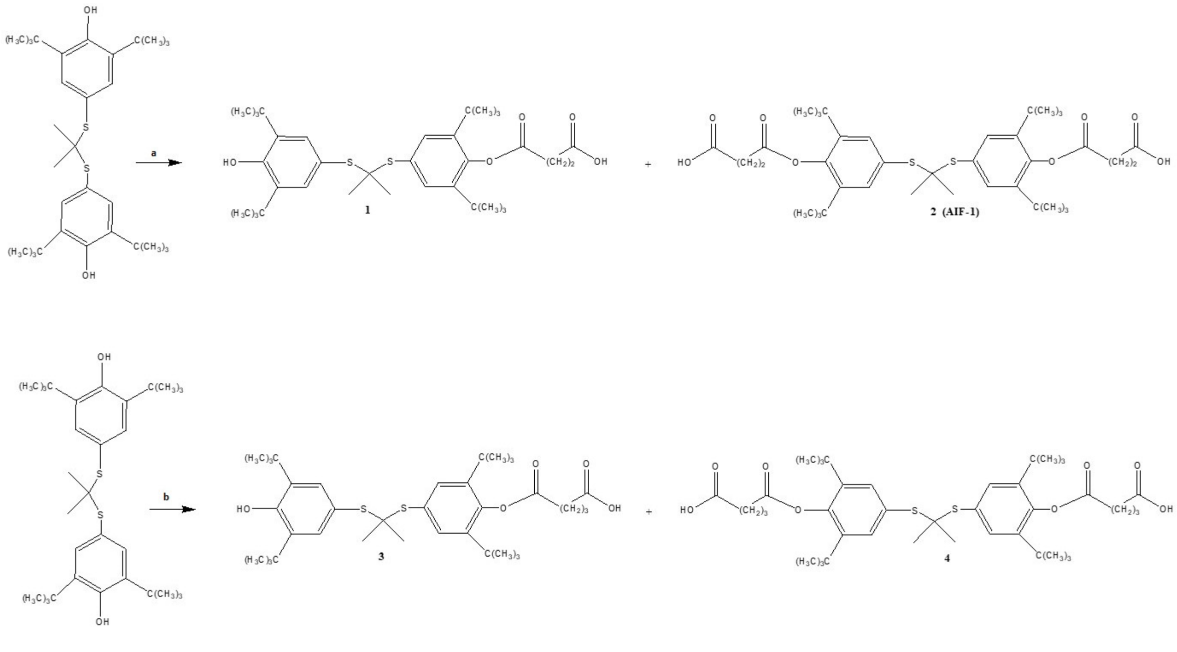

Scheme S1. Reagents and conditions (a) NaH 60%, succinic anhydride, THF; (b) NaH 60%, glutaric anhydride, THF.

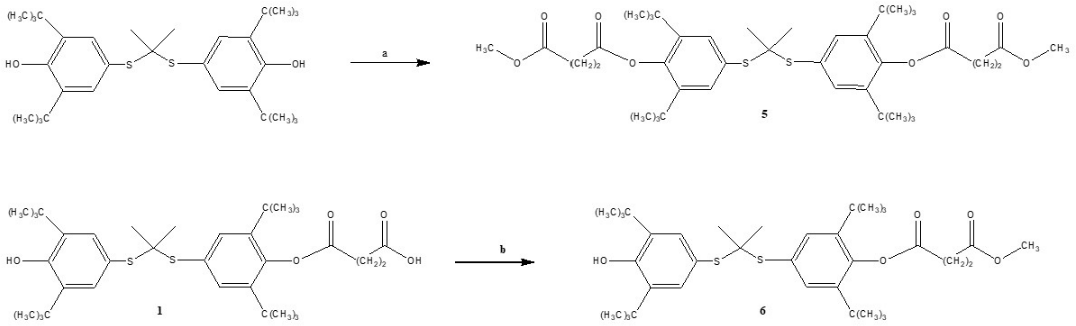

Scheme S2. Reagents and conditions (a) NaH 60%, methyl-3-(chlorocarbonyl)propanoate; (b) Triphenylphospine, diisopropyl azodicarboxylate (DIAD), MeOH, THF.

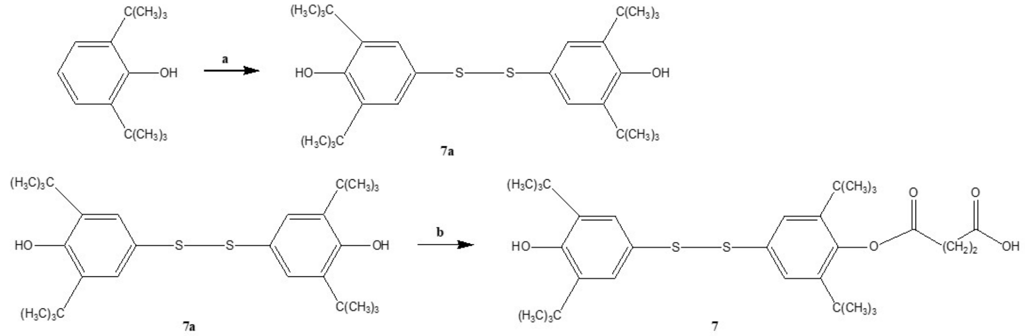

Scheme S3. Reagents and conditions (a) S_2Cl_2 Toluene; (b) NaH 60%, succinic anhydride, THF.

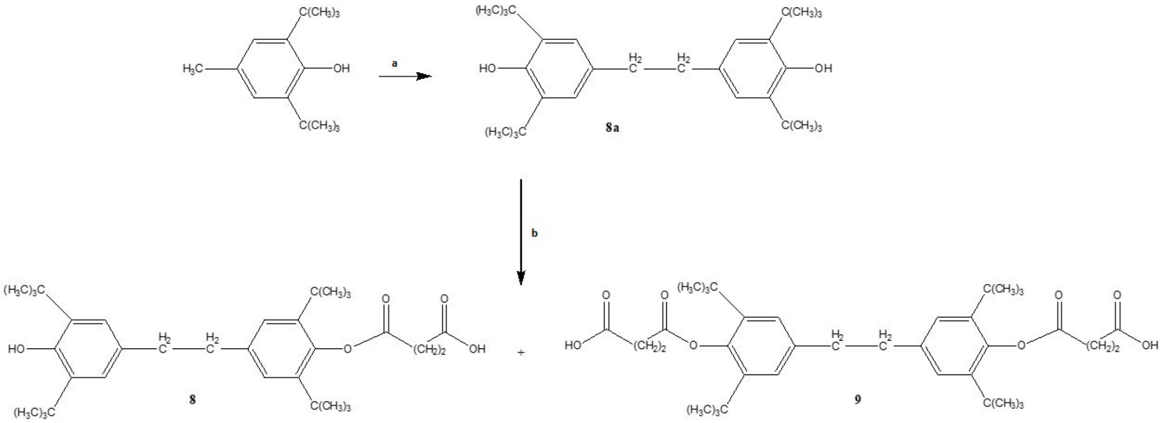

Scheme S4. Reagents and conditions (a) $K_3[Fe(CN)_6]$, KOH, toluene 60°C; (b) NaH 60%, succinic anhydride, THF.

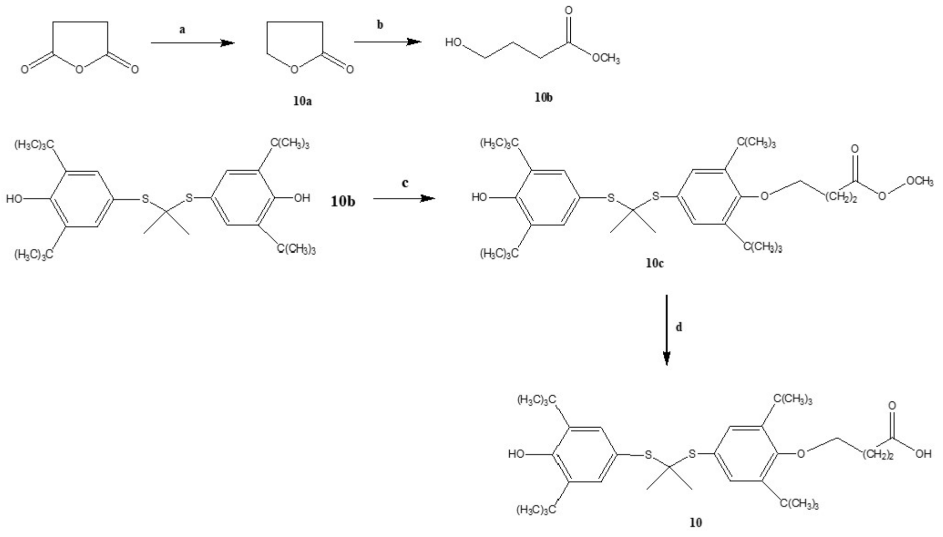

Scheme S5. Reagents and conditions (a) $LiBH_4$ THF (b) Amberlist-1 5, MeOH; (c) Triphenylphospine, diisopropyl azodicarboxylate (DIAD), THF; (d) KOH 15%, EtOH.

4-[2,6-ditert-butyl-4-[-(3,5-ditert-butyl-4-hydroxyphenyl)sulfanylpropan-2-ylsulfanyl]phenoxy]-4-oxobutanoic acid (1) and **4-[2,6-ditert-butyl-4-[2-[3,5-ditert-butyl-4-(3-carboxypropanoyloxy)phenyl]sulfanylpropan-2-ylsulfanyl]phenoxy]-4-oxobutanoic acid (2)**

A protocol reported in the literature was followed [48] : to a stirred suspension of probucol (0.965 mmol) and NaH 60% (w/w dispersion in mineral oil) (5.00 mmol) in anhydrous THF (10 mL) under N_2 was added dropwise succinic anhydride (5.8 mmol) previously dissolved in anhydrous THF (12 ml). The mixture was stirred and kept under nitrogen atmosphere overnight. After acidification with 2N HCl, the solvent was removed under reduced pressure and the resulting suspension was extracted with ethyl acetate (3 x 100 ml). The organic extracts were washed with water, brine and dried with anhydrous Na_2SO_4. Evaporation of the solvent under reduced pressure yielded a viscous oil that was purified by flash chromatography [SiO_2]; CH_2Cl_2:HCOOH:C_2H_5OH from 99.87:0.13:0.5 (200 ml) to 98.75:0.25:1 (200 ml). Afforded to a first fraction compound **1** and a second fraction compound 2**.**

4-[2,6-ditert-butyl-4-[-(3,5-ditert-butyl-4-hydroxyphenyl)sulfanylpropan-2-ylsulfanyl]phenoxy]-4-oxobutanoic acid (1)

The crude product was recrystallized from ethanol-water to give **1** (0.132 g 22%) as a white solid; ^{1}H-NMR (400 MHz $CDCl_3$) δ = 1.36 (S,18H, $(CH_3)_3$), 1.46 (s,18H, $(CH_3)_3$), 1.48 (s, 6H, CH_3), 2.81 (t, 2H, J = 7.3 CH_2), 3.01 (t, 2H, J = 7.3 CH_2), 5.39 (s, 1H, OH); 7.47 (s, 2H, Ar), 7.67 (s, 2H, Ar); MS (ESI) calcd. for $C_{35}H_{52}O_5S_2$ 616,91 found 617 [M-1$^-$]$^-$.

4-[2,6-ditert-butyl-4-[2-[3,5-ditert-butyl-4-(3-carboxypropanoyloxy)phenyl]sulfanylpropan-2-ylsulfanyl]phenoxy]-4-oxobutanoic acid(2) Compound **2** was recrystallized from ethanol-water to afford a white solid. (0.120 g 17%) ^{1}H-NMR

(400 MHz $CDCl_3$) δ = 1.30 (S,36H, $(CH_3)_3$), 1.50 (s, 6H, CH_3), 2.80 (t, 2H, J = 7.3 CH_2), 3.00 (t, 2H, J = 7.3 CH_2), 7.64 (s, 4H, Ar); MS (ESI) calcd. for $C_{39}H_{56}O_8S_2$ 716.34 found 739.4 [M+Na].

5-[4-([41]sulfanyl)-2,6-di-tert-butylphenoxy]-5-oxopentanoic (3) and **4-[2,6-ditert-butyl-4-[2-[3,5-ditert-butyl-4-(carboxybutanoyloxy)phenyl]sulfanylpropan-2-ylsulfanyl]phenoxy]-5-oxopentanoic acid (4)**

A protocol reported in the literature was followed [48]: to a stirred suspension of probucol (0.965 mmol) and NaH 60% (w/w dispersion in mineral oil) (5.00 mmol) in anhydrous THF (10 mL) under N_2 was added dropwise glutaric anhydride (5.8 mmol) previously dissolved in anhydrous THF (12 ml). The mixture was stirred and kept under nitrogen atmosphere overnight. After acidification with 2N HCl, the solvent was removed under reduced pressure and the resulting suspension was extracted with ethyl acetate (3 x 100 ml). The organic extracts were washed with water, brine and dried with anhydrous Na_2SO_4. Evaporation of the solvent under reduced pressure yielded a viscous oil that was purified by flash chromatography [SiO_2, CH_2Cl_2 (200 ml); CH_2Cl_2:HCOOH:C_2H_5OH from 99.87:0.13:0.5 (200 ml) to 98.75:0.25:1 (200 ml). afforded to a first fraction which compound **3** and a second fraction compound **4.**

5-[4-([41]sulfanyl)-2,6-di-tert-butylphenoxy]-5-oxopentanoic (3) The crude product was recrystallized from ethanol-water to give **3** (0.130 g 29%) as a white solid; ^{1}H-NMR (400 MHz $CDCl_3$) δ = 1.35 (S,18H, $(CH_3)_3$), 1.45 (s,18H, $(CH_3)_3$), 1.48 (s, 6H, CH_3), 2.09 (quint, 2H, J = 7.3 CH_2), 2.57 (t, 2H, J = 7.3 CH_2), 2.76 (t, 2H, J = 7.3 CH_2), 5.39 (s, 1H, OH); 7.46 (s, 2H, Ar), 7.64 (s, 2H, Ar); MS (ESI) calcd. for $C_{36}H_{54}O_5S_2$ 630,94 found 629 $[M-1^-]^-$.

4-[2,6-ditert-butyl-4-[2-[3,5-ditert-butyl-4-(carboxybutanoyloxy)phenyl]sulfanylpropan-2-ylsulfanyl]phenoxy]-5-oxopentanoic acid (4) Compound 4 was recrystallized from ethanol-water to afford a white solid. (0.274 g 53%) ^{1}H-NMR (400 MHz $CDCl_3$) δ = 1.35 (S,36H, $(CH_3)_3$), 1.51 (s, 6H, CH_3), 2.11 (quint, 4H, 4H, J = 7.3 CH_2), 2.57 (t, 4H, J = 7.3 CH_2), 2.77 (t, 4H, J = 7.3 CH_2), 7.64 (s, 4H, Ar); MS (ESI) calcd. for $C_{41}H_{60}O_8S_2$ 745.04 found 768.06 [M+Na].

4-[2,6-ditert-butyl-4-[2-(3,5-ditert-butyl-4-(3-carboxypropanoyloxy)phenyl]sulfanylpropan-2-ylsulfanyl]phenoxy]-4-oxobutanoic acid dimethyl ester (5)

To a stirred suspension of probucol (0.965 mmol) and NaH 60% (w/w dispersion in mineral oil) (5.00 mmol) in anhydrous THF (10 mL) under N_2 was added methyl-3-(chlorocarbonyl) propanoate (4.0 mmol) previously dissolved in anhydrous THF (2 ml). The mixture was stirred and kept under nitrogen atmosphere overnight. After acidification with 2N HCl, the solvent was removed under reduced pressure and the resulting suspension was extracted with ethyl acetate (3 x 100 ml). The organic extracts were washed with water, brine and dried with anhydrous Na_2SO_4. Evaporation of the solvent under reduced pressure yielded a viscous oil that was purified by flash chromatography [SiO_2,CH_2Cl_2 (200 ml) with CH_2Cl_2:C_2H_5OH 99.5:0.5 as eluent afforded to compound **5** which was recrystallized from ethanol affording a white solid. (0.600 g 83%) ^{1}H-NMR (400 MHz $CDCl_3$) δ = 1.34 (S, 36H, $(CH_3)_3$), 1.49 (s, 6H, CH_3), 2.74 (t, 2H, J = 7.3 CH_2), 3.02, 3.71 (s, 6H, CH_3O), 7.63 (s, 4H, Ar); MS (ESI) calcd. for $C_{40}H_{60}O_8S_2$ 745,04 found 768 [M+Na].

4-[2,6-ditert-butyl-4-[2-(3,5-ditert-butyl-4-hydroxyphenyl)sulfanylpropan-2-ylsulfanyl]phenoxy]-4-oxobutanoic acid methyl ester (6)

Triphenylphosphine (0.329 mmol) was dissolved in anhydrous THF (10 ml) and cooled to 0°C. To the stirred solution under nitrogen was added dropwise a solution of diisopropyl azodicarboxilate (DIAD) (0.324 mmol) in anhydrous THF (3ml), keeping the temperature at 0°C. After the addition was complete, the reaction mixture was warmed to room temperature and then a solution of **1** (0.259 mmol) and methyl alcohol (0.259 mmol) in anhydrous THF (5 ml) was added dropwise. After stirring overnight at room temperature, the solvent was removed under reduced pressure and the residue was purified by flash chromatography [SiO_2, CH_2Cl_2:n-hexane from 20:80 (200 ml), to 40:60 CH_2Cl_2: n-hexane (200 ml)]. The crude product was recrystallized from ethanol-water to give **4** (0.060 g, 52%) as a white solid; ^{1}H-NMR (300 MHz $CDCl_3$ δ = 1.35 (s, 18H, $(CH_3)_3$), 1.45 (s, 18H, $(CH_3)_3$), 1.47 (s, 6H, CH_3), 2.75 (t, 2H, *J* = 7.0 CH_2), 3.02 (t, 2H, *J* = 7.0 CH_2), 3.70 (s, 3H, CH_3), 3.72 (s, 3H, CH_3O), 5.38 (s, 1H, OH), 7.46 (s, 2H, Ar), 7.64 (s, 2H, Ar); MS (ESI) calcd for $C_{36}H_{54}O_5S_2$ 630.94 found 629 $[M-1^-]^-$.

Bis(3,5-ditert-butyl-4-hydroxyphenyl) disulfide (7a)

Compound **7a** was synthesized following a described procedure [50] Stocker R. O. Patent WO2006/63408 2006 starting from 2,6-ditertbutylphenol.

4-(2,6,-ditert-butyl-4-((3,5-ditert-butyl-4-hydroxyphenyl)-4'-(1-carboxy-3-oxo-propan-2,6-ditert-butyl-4-((3,5-ditert-butyl-4-hydroxyphenyl)-disulfanyl)phenoxy)-4-oxobutanoic acid (7)

Compound **7** was synthesized following a described procedure [49] starting from compound **7a** and succinic anhydride.

1,2-Bis(3,5-ditert-butyl-4-hydroxyphenyl)ethane (8a)

Compound **8a** was prepared following a described procedure [51] starting from 2,6-di-tert-butyl-4-methylphenol.

4--4-oxobutanoic acid (8) and 4-[2,6-ditert-butyl-4-[2-[3,5-ditert-butyl-4-(3-carboxypropanoyloxy)phenyl]ethyl]phenoxy]-4-oxobutanoic acid (9)

To a stirred suspension of NaH 60% (w/w dispersion in mineral oil) (2.642 mmol) and **8a** (1.360 mmol) in anhydrous THF (15 mL) under N_2 was added dropwise succinic anhydride (1.50 mmol) previously dissolved in anhydrous THF (4 ml). The mixture was stirred and kept under nitrogen atmosphere overnight. After acidification with 2N HCl the solvent was removed under reduced pressure and the resulting suspension was extracted with ethyl acetate (3 × 50 ml). The organic extracts were washed with water, brine and dried with anhydrous Na_2SO_4. Evaporation of the solvent under reduced pressure yielded a white solid that was purified by flash chromatography [SiO_2] CH_2Cl_2:HCOOH:C_2H_5OH from 99.87:0.13:0.5 (200 ml) to 98.75:0.25:1 (200 ml). Afforded to a first fraction compound **8** and a second fraction compound **9.**

4-[43]-4-oxobutanoic acid (8) Compound **8** was recrystallized from ethanol-water to give a white solid (0.182 g 25%) as a white solid ^{1}H-NMR (400.13 MHz $CDCl_3$) δ = 1.33 (S,18H, $(CH_3)_3$), 1.45 (s,18H, $(CH_3)_3$), 2.81 (t, 2H, *J* = 7.2. CH_2), 2.87 (s, 4H, CH_2), 3.00 (t, 2H, *J* = 7.3 CH_2), 5.06 (s,1H, OH); 6.99 (s,2H, Ar), 7.12 (s, 2H, Ar); MS (ESI) calcd. for $C_{34}H_{50}O_5$ 538.76 found 537 $[M-1^-]^-$.

4-[2,6-ditert-butyl-4-[2-[3,5-ditert-butyl-4-(3-carboxypropanoyloxy)phenyl]ethyl]phenoxy]-4-oxobutanoic acid (9)

Compound **9** was recrystallized from ethanol-water to give a white solid (0.114 g 26%) as a white solid ^{1}H-NMR (400.13

MHz $CDCl_3$) $\delta = 1.29$ (S,36H, $(CH_3)_3$), 2.79 (t, 2H, $J = 7.2$. CH_2), 2.90 (s, 4H, CH_2), 2.98 (t, 2H, $J = 7.3$ CH_2), 7.09 (s, 4H, Ar); MS (ESI) calcd. for $C_{38}H_{54}O_8$ 638.83 found 638.83$[M+Na1^-]^+$.

Dihydrofuran-2(3*H*)-one (10a)

Compound **10 a** was synthesized following a described procedure [52]. Improved procedure for lithium borohydride reduction of cyclic anhydrides to lactones in tetrahydrofuran. Heterocycles, 18, 1982, 131-135 starting from succinic anhydride.

4-hydroxybutanoic acid methyl ester (10b)

Compound **10 b** was synthetized following a described procedure [53] starting from compound **10 b** and methylic alcol.

4-[-(sulfamyl) -2,6-ditert-butylphenoxy] butanoic acid methyl ester (10 c)

Triphenylphosphine (2.40 mmol) was dissolved in anhydrous THF (20 ml) and cooled to 0°C. To the stirred solution under nitrogen was added dropwise a solution of diisopropyl azodicarboxilate (DIAD) (2.38 mmol) in anhydrous THF (4ml), keeping the temperature at 0°C. After the addition was complete, the reaction mixture was warmed to room temperature and then a solution of **5c** (1.890 mmol) and probucol (0.94 mmol) in anhydrous THF (20 ml) was added dropwise. After stirring overnight at room temperature, the solvent was removed under reduced pressure and the residue was purified by flash chromatography [SiO_2, CH_2Cl_2:n-hexane from 90:10 (200 ml) to 100% CH_2Cl_2: (100 ml)] furnishing a viscous oil (0.116 g, 23%); ^{1}H-NMR (300 MHz $CDCl_3$ $\delta = 1.42$ (s, 18H, $(CH_3)_3$), 1.45 (s, 24H, CH_3), 2.22 (p, 2H, $J = 7.3$ CH_2), 2.50 (t, 2H, $J = 7.3$ CH_2), 3.70 (s, 3H,CH_3), 3.75 (t, 2H, $J = 7.2$ CH_2), 5.38 (s, 1H, OH), 7.46 (s, 2H, Ar), 7.54 (s, 2H, Ar), 8.05 (d, 2H, $J = 8.4$ Ar). MS (ESI) calcd for $C_{36}H_{56}O_4S_2$ 616.56 found 617 $[M-1^-]^-$.

4-[4-([45]sulfanyl)-2,6-di-*tert*-butylphenoxy]butanoic acid (10)

To a solution of **10C** (0.650 mmol) in ethanol (75 ml) was added a solution of sodium hydroxide 15% w/v (20 ml) and the mixture was stirred at room temperature overnight. Ethanol was removed under vacuum and the solution was acidified with concentrated hydrochloric acid until a precipitate was formed. The solid was filtered off under vacuum and washed with distilled water.

The crude product was recrystallized from ethanol-water to give **10** (0.335 g, 85%) as a white solid. Mp 157-160 °C; ^{1}H-NMR (300 MHz DMSO-d6) $\delta = 1.39$ (s, 6H, CH_3); 1.43-144 (m, 36H, $(CH_3)_3$), 2.17 (p, 2H, $J = 7.3$ CH_2), 2.45 (t, 2H, $J = 7.3$ CH_2), 3.79 (t, 2H, $J = 7.3$ CH_2), 4.87 (s, 1H, OH), 7.42 (s,2H, Ar), 7.56 (s, 2H, Ar); MS (ESI) calcd for $C_{35}H_{54}O_4S_2$ 602.93 found 375 $[M-1]^-$. MS (ESI) calcd. for $C_{35}H_{54}O_4S_2$ 602.93 found 601 $[M-1^-]^-$. Anal. calcd for $C_{35}H_{54}O_4S_2$: C, 69.72; H, 9.03. Found: C, 69.72; H, 9.30.

Result

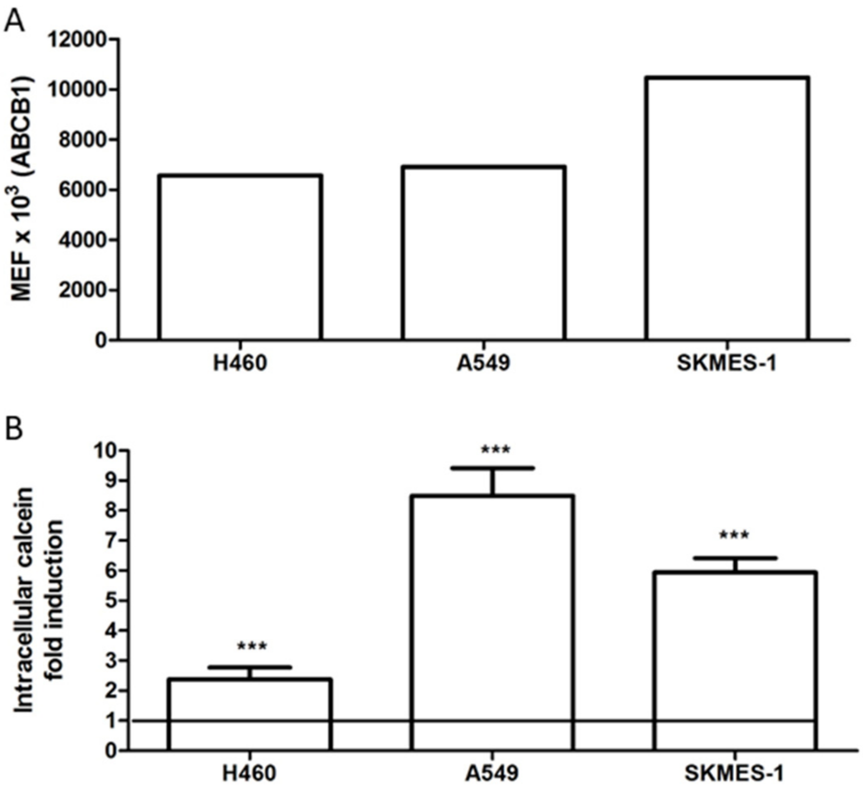

Figure S1: Characterization of ABCB1 expression and activity in NSCLC cell lines. (**A**) ABCB1 protein levels on cell surface were quantified by flow-cytometry and expressed as molecular equivalent of fluorochrome (MEF) as described in the Methods Section. (**B**) Cells were loaded with 1 μM calcein AM in the presence of PSC833. After 4 hours, calcein AM was removed and its fluorescence was determined by luminometer. Relative ABCB1 activity was defined as the ratio of calcein accumulation per μg of protein between PSC833 treated and untreated cells and was expressed as fold increase of inhibition activity. Results are representative of at least three independent experiments. Statistical significance was calculated by *t* Test (*** $p < 0.001$).

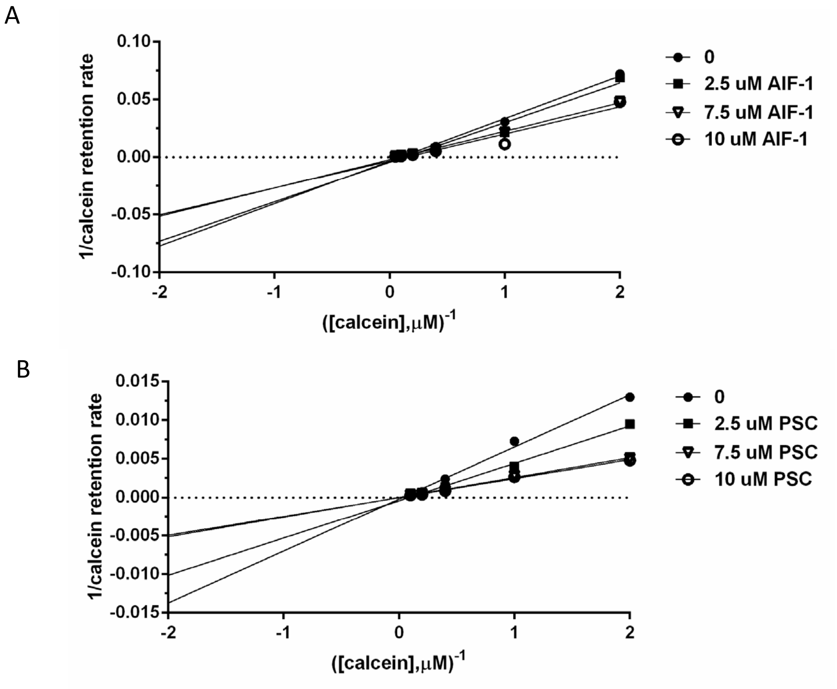

Figure S2: Determination of mode of inhibition by AIF-1 compound in SKMES-1 cell line. Lineweaver-Burk plot of ABCB1-mediated Calcein transport at concentrations of 0.5, 1, 2.5, 5, 10, 20 μM. AIF-1 compound (**A**) and PSC833 (**B**) were used at concentrations of 2.5, 7.5 and 10 μM. Depicted are representative experiments out of three independent experiments.

A

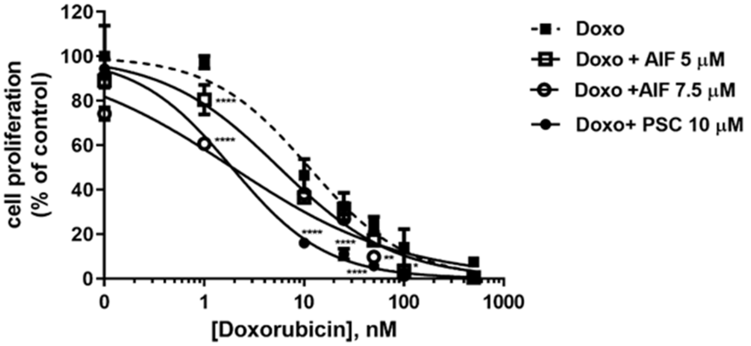

B

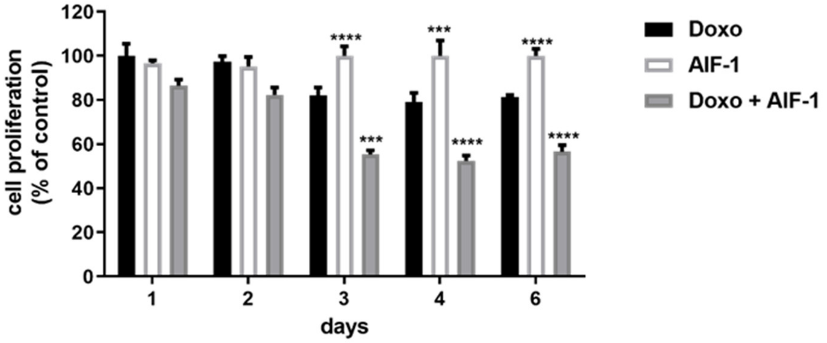

Figure S3: Concentration- and time-dependent efficacy of AIF-1. (**A**) SKMES-1 cells were treated with increasing concentration of doxorubicin, in the presence or absence of AIF-1 (5 or 7.5 μM) or PSC833 (10 μM). After 72 hours crystal violet assay was performed, and absorbance was measured at 570 nm. Data are expressed as percent cell growth versus control cells (mean ± SD). Statistical significance was calculated by one way ANOVA with Bonferroni's post hoc analysis. (* $p < 0.05$, ** $p < 0.01$, **** $p < 0.0001$ vs doxorubicin treatment at each concentration). (**B**) SKMES-1 cells were treated with 10 nM doxorubicin and 7.5 M AIF-1. After 1,2,3,4 and 6 days crystal violet assay was performed, and absorbance was measured at 570 nm. Data are expressed as percent cell growth versus control cells (mean ± SD). Statistical significance was calculated by one way ANOVA with Bonferroni's post hoc analysis. (*** $p < 0.001$, **** $p < 0.0001$ vs doxorubicin treatment).

References

48. Meng, C.Q.; Somers, P.K.; Rachita, C.L.; Holt, L.A.; Hoong, L.K.; Zheng, X.S.; Simpson, J.E.; Hill, R.R.; Olliff, L.K.; Kunsch, C.; et al. Novel phenolic antioxidants as multifunctional inhibitors of inducible VCAM-1 expression for use in atherosclerosis. *Bioorg Med Chem Lett* **2002**, *12*, 2545-2548, doi:10.1016/s0960-894x(02)00516-4.
49. Parker, R.A.; Barnhart, R.L.; Chen, K.S.; Edwards, M.L.; Matt, J.E.; Rhinehart, B.L.; Robinson, K.M.; Vaal, M.J.; Yates, M.T. Antioxidant and cholesterol lowering properties of 2,6-DI-t-butyl-4-[(dimethylphenylsilyl)methyloxy]phenol and derivatives: A new class of anti-atherogenic compounds. *Bioorganic & Medicinal Chemistry Letters* **1996**, *6*, 1559-1562, doi:10.1016/S0960-894X(96)00273-9.
50. STOREY, J., Mervyn, David SINCLAIR, James, Peter MARSHALL, Colin TAN, Han Wan WISCHIK, Claude, Michel. Methods of chemical synthesis and purification of diaminophenothiazinium compounds including methylthioninium chloride (MTC). 2006.
51. Cook, C.D.; Nash, N.G.; Flanagan, H.R. Oxidation of Hindered Phenols. III. The Rearrangement of the 2,6-Di-t-butyl-4-methylphenoxy Radical. *Journal of the American Chemical Society* **1955**, *77*, 1783-1785, doi:10.1021/ja01612a019.
52. Narasimhan, S. Improved procedure for lithium borohydride reduction of cyclic anhydrides to lactones in tetrahydrofuran. . **1982**, *18*, 131-135, doi:DOI: 10.3987/S(B)-1982-01-0131.
53. Anand, R.C.; Selvapalam, N. A Convenient and Mild Procedure for the Preparation of Hydroxyesters from Lactones and Hydroxyacids. *Synthetic Communications* **1994**, *24*, 2743-2747, doi:10.1080/00397919408010590.
